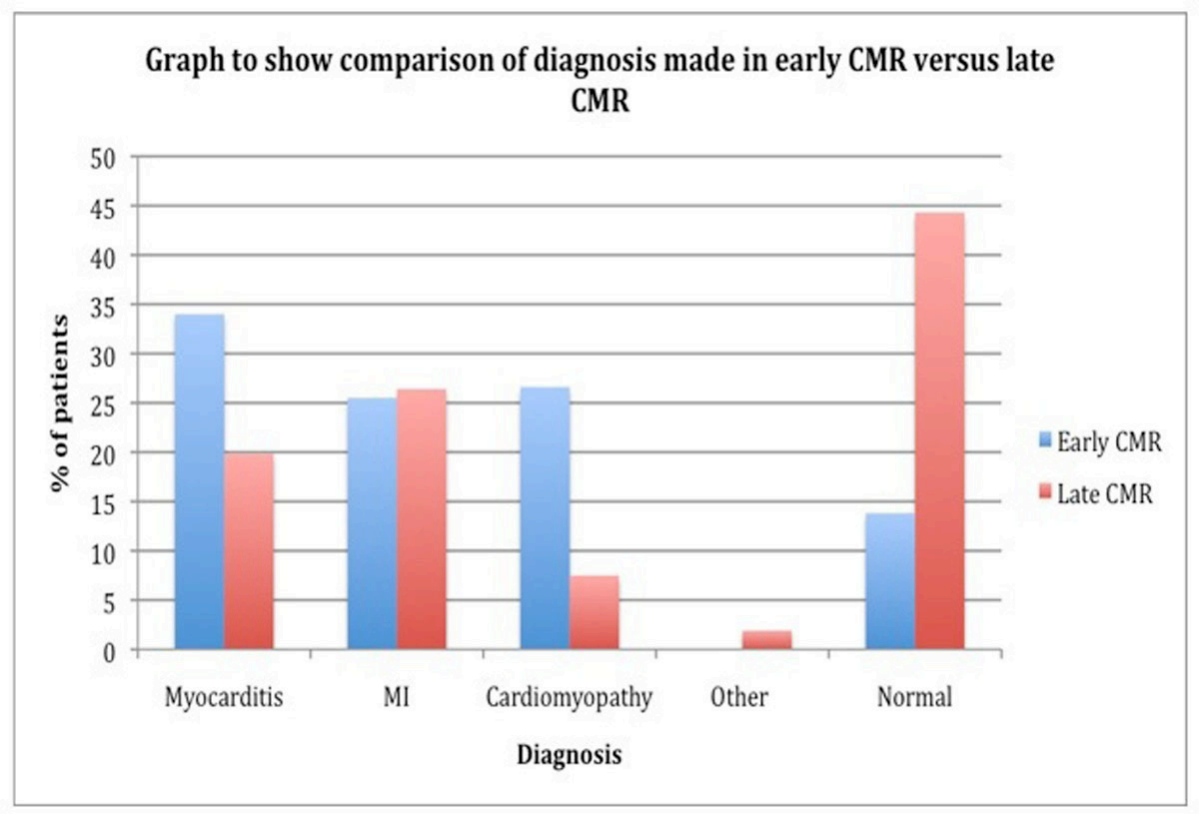# Improved diagnostic role of CMR in acute coronary syndromes and unobstructed coronary arteries: the importance of time-to-CMR

**DOI:** 10.1186/1532-429X-17-S1-O87

**Published:** 2015-02-03

**Authors:** Amardeep Ghosh Dastidar, Priyanka Singhal, Jonathan C Rodrigues, Nauman Ahmed, Alberto Palazzuoli, Mandie Townsend, Angus K Nightingale, Tom Johnson, Julian Strange, Andreas Baumbach, Chiara Bucciarelli-Ducci

**Affiliations:** 1 NIHR Cardiovascular Biomedical Research Unit, Bristol Heart Institute, Bristol, UK; 2 University of Bristol, Bristol, UK

## Background

Acute coronary syndrome (ACS) still remains one of the leading causes of mortality and morbidity. In the literature 7-15% of patients with ACS have non obstructive coronary artery disease. In these patients CMR can identify different underlying etiologies, mainly myocarditis, myocardial infarction (MI) with spontaneous recanalization/embolus or Tako-Tsubo cardiomyopathy. However the diagnostic pick-up rate of these aetiologies by CMR is highly variable in the literature and patients are not consistently scanned in the same time window.

## Aim

To evaluate the diagnostic role of performing CMR "early" (< 2 weeks from presentation) versus "late" (>2 weeks from presentation) in patients with troponin positive ACS and unobstructed coronaries.

## Methods

In this retrospective observational study, performed at a large cardiothoracic tertiary centre in the South-West of England, data were collected on consecutive patients with troponin positive ACS and unobstructed coronaries, referred for a CMR (September 2011 to July 2014). CMR was performed on a 1.5T scanner (Avanto, Siemens) using a comprehensive protocol that included long- and short-axis cines, T2 weighted STIR and early and late gadolinium enhancement. Each scan was reported by a consultant with >10yrs CMR experience.

## Results

204 consecutive patients (mean age 55yrs) were included in the analysis (51% males). The median time interval between presentation and CMR was 20 days (range 1-150days).

An "early" CMR was performed in 96 patients (median 6days and range 1-14days) and 108 patients underwent a "late" CMR scan(median 41 days and range 15-150days). Overall, a cause for the troponin rise was found in 70% of patients, whilst in 30% no CMR abnormalities were detected. The diagnostic pick up rate significantly improved when the scan was done early: 82% vs 54% when CMR performed "late" (p<0.0001). Myocarditis was the most common diagnosis in the early arm (34%) whereas reperfused MI in the late group (26%).

## Conclusions

In a large cohort of patients with troponin positive ACS and unobstructed coronary arteries CMR was able to establish a final diagnosis in overall 70% of patients (and found to be normal in 30%). The diagnostic value of CMR in patients with troponin positive ACS and unobstructed coronaries improves significantly when carried out within 2 weeks from acute presentation. In these patients establishing a final diagnosis has a definite impact in patient management and hence CMR should be offered in a specified time window from presentation.

## Funding

This study was funded by the National Institute for Health Research Biomedical Research Unit in Cardiovascular Disease at the University Hospitals Bristol NHS Foundation Trust and the University of Bristol.

**Figure 1:**